# The ontogeny of myometrial stem cells in OCT4-GFP transgenic mouse model

**DOI:** 10.1186/s13287-018-1079-7

**Published:** 2018-11-29

**Authors:** Soumia Brakta, Aymara Mas, Ayman Al-Hendy

**Affiliations:** 1 Department of Obstetrics and Gynecology, University of Augusta, Augusta, GA 30912 USA; 2 Reproductive Medicine Research Group, La Fe Research Institute, Valencia, Spain; 3 Department of Obstetrics and Gynecology, University of Illinois at Chicago (UIC), Chicago, IL 60612 USA

**Keywords:** Somatic stem cells (SSCs), Myometrium, OCT4-GFP transgenic mice, CD44, Nanog

## Abstract

**Background:**

Myometrium, the muscular wall of the uterus, is an active organ markedly remodeled during a woman’s reproductive life, especially during pregnancy. Different studies using the 5-bromo-2′-deoxyuridine and side population methods in murine and human myometrium have suggested the presence of somatic stem cells in this tissue because of its remarkable regenerative capacity. Recently, our group has developed a surface-marker (Stro1/CD44)-specific approach to isolate and characterize myometrial somatic stem cells (SSCs) from humans and rats.

**Objective:**

In this study, we aimed to identify and localize the putative myometrial stem cell population in the murine uterus by using the specific surface markers, Nanog/CD44.

**Methods:**

Uteri from OCT4-GFP transgenic mice at different early-life time points were analyzed via single and double immunohistochemistry to co-localize myometrial stem cell marker CD44 with other general stemmness markers, e.g., Nanog and Oct-4. Finally, we correlated the frequency of myometrial stem cells in vivo with the expression of sex steroid hormone receptors, estrogen receptor α (ERα), and progesterone receptors A and B (PR A&B).

**Results:**

Nanog^+^/CD44^+^ stem cells were present in murine myometrium. Both stem cell markers were shown to co-localize with Oct-4 expression. Time-course experiments demonstrated that their percentages were significantly lower at the pre-sexual age of 1 week than at the sexually mature ages of 3 to 24 weeks. Importantly, both ERα and PR A&B were abundantly expressed in the myometrium at ages 1, 3 and 4 weeks.

**Conclusions:**

We demonstrated that murine CD44^+^ myometrial cells have features of somatic stem cells with the expression of typical undifferentiated markers. Furthermore, our results suggest that myometrial stem cells are sex steroid hormone dependent, likely via paracrine pathway, and increase in numbers with reproductive maturity and rise in serum estrogen and progesterone levels around 3 weeks of age in mice. The abundance and early onset expression of ER/PR emphasize the vulnerability of neonatal myometrium to environmental endocrine disruptors which can potentially lead to permanent reprograming and adult onset of myometrial disorders such as uterine fibroids.

**Electronic supplementary material:**

The online version of this article (10.1186/s13287-018-1079-7) contains supplementary material, which is available to authorized users.

## Background

After embryonic development, tissue-specific stem cells called adult or somatic stem cells (SSCs) remain throughout the body for life. These adult SSCs are master cells that, through asymmetric division, retain their ability to self-renew while producing daughter cells that are the functional units of that tissue [1, 2]. The daughter cells completely differentiate to support tissue repair and remodeling contributing to the structural and functional maintenance of their tissue of residence [1]. Tissue-specific stem cells have been identified in multiple tissues and organs including human and murine myometrium [3, 4]. Different characteristics have been used to identify stem cell lines such as cell clonogenic efficiency, in vitro differentiation, expression of stemness markers, and in vivo tissue regeneration [5–8]. Specifically, mesenchymal stem cells (MSCs) express specific embryonic stem cell genes like Oct-4 and Nanog, transcription factors which determine the embryonic stem cell self-renewal and differentiation [9].

The human uterus consists of three tissue layers: endometrium, myometrium, and perimetrium [10]. The myometrium is the smooth muscle layer of the uterus, oftentimes characterized by its ability to remodel and regenerate during and after pregnancy [11, 12]. These unique properties suggest the presence of myometrial stem cells that tightly regulate myometrial growth [3, 4]. Similarly, tumor-initiating cells are a subset of cells within a tumor cell population, which also through asymmetric division retain their ability to sustain tumors [1, 13]. Uterine leiomyomas, also called uterine fibroids, are monoclonal tumors of the myometrium that likely originate from a single altered and transformed somatic stem cell of the myometrium followed by expansion and propagation in a steroid-dependent manner [12–14]. Stem cells derived from leiomyoma tissue, but not myometrium, carry a mediator complex subunit 12 (MED 12) mutation in the majority of leiomyoma lesions [15]. It is thus hypothesized that the transformation of a myometrial stem cell into a mutated stem cell leads to the formation of a leiomyoma tumor progenitor cell that, after further expansion, gives rise to leiomyoma [2, 4, 8, 16]. The transformation of a normal myometrial stem cell into a leiomyoma-forming stem cell is likely a result of a complex process entailing multiple insults to the myometrial stem cell including hypoxic niche, altered epigenome, and abnormal estrogen signaling.

Our group has recently identified two specific cell surface markers (CD44 & Stro1) as human [17] and rat [18, 19] myometrial stem cell markers. CD44 is a generic name for a complex set of cell surface glycoproteins involved in cell proliferation, differentiation, and migration [20]. Even though human and rat myometrial stem cells have been well characterized by our group and others [17–19, 21, 22], little has been published on mouse myometrial stem cells since 2007 when Dr. Szotek suggested myometrial labeling-retaining cells (LCRs) are putative myometrial stem cells. In this work, we evaluate the utility of specific cell surface markers to identify myometrial stem cells in mice at different age points as well as determine the effect of initiation of steroidogenesis on the frequency of these stem cells.

## Materials and methods

### Mouse tissues

Female mice, B6, CBA-Tg (Pou-5 fl-EGFP) 2Mnn/j mouse strains, 1 week to 24 weeks of age were purchased from Jackson laboratory (Sacramento, CA). These mice were homozygous for the Pou5fl/OCT4 transgenic insert and expressed enhanced green fluorescent protein (EGFP) in the uterus under the control of POU domain, class 5, transcription factor 1 (Oct-4) promoter, and distal enhancer. Primordial germ cell-specific markers, alkaline phosphatase II, and stage-specific embryonic antigen are co-expressed in EGFP-positive cells. Pou5fl or Oct-4 expression indicates the totipotent cell lineage [23]. Mice homozygous for the transgenic insert were reported as viable, fertile, and normal in size; they did not display gross physical or behavioral abnormalities. Uterine tissue was collected at ages 1, 3, 4, 8, 12, and 24 weeks (at least 4 mice per age group).

All animal procedures described in this report have been approved by Augusta University’s Institutional animal care and utilization committee (IACUC).

### Immuno-co-localization with undifferentiated markers

Immunohistochemistry (IHC) and immunofluorescence approaches were performed, and myometrial stem cells were visualized under green fluorescence. To co-localize the identified Oct-4-positive myometrial stem cells, mice tissue was co-stained with CD44 antibody and Nanog.

Tissue blocks were deparaffinized in a Leica XL Autostainer (Leica Inc., Buffalo Grove, IL), and antigen retrieval was performed with Antigen Unmasking Solution (Vector Labs, Burlingame, CA). Following pre-incubation of tissue with Blocking Buffer (10% normal goat serum, 1% BSA, 0.5% Triton X-100) for 1 h at room temperature, primary antibody was added to Blocking Buffer and incubation was continued overnight at room temperature. Primary antibodies were diluted as follows: CD44 at 1/250 and Nanog at 1/200 (Additional file 1: Table S1). Slides were then washed three times in PBS and treated with fluorescein or Texas Red conjugated secondary antibodies (Vector Labs) for 1 h at room temperature. Following three washes in PBS, slides were cover slipped with mounting medium containing Dapi (Vector Labs).

### Cell counting

Four-micrometer sections were stained with the indicated antibodies and counterstained with Mayer’s hematoxylin or DAPI, respectively, to visualize all cells present in the section and perform the enumeration of positive cells. Cells were counted by using NIH ImageJ software. At least, 200 cells were counted for each time point against DAPI and hematoxylin-stained nuclei. Labeling index and percentages were determined by the coefficient between the positive cells for the specific antibodies and the total number of cells in each slide determined by hematoxylin nuclear staining or DAPI (fluorescent technique). Three random high-power fields were selected from each mouse age time point, and the average stem cell percentage was determined. Data was expressed as mean and standard errors.

### Histological examination for sex steroid hormone receptors

To study the possible effect of ovarian sex steroids on the quantity of stem cells, we evaluated myometrial expression of estrogen receptor α (ERα) and progesterone receptors A and B (PR A&B) at ages 1, 3, and 4 weeks using inverted microscopy. Blocks of uterine tissue at the indicated age time points were deparaffinized and prepared as stated above. Primary antibodies were diluted as follows: estrogen receptor at 1/250 and progesterone receptor at 1/250 (Additional file 1: Table S1). Slides were visualized under inverted microscopy for presence of brown staining indicating presence of the receptor. Appropriate positive and negative controls were obtained for comparison using standard techniques according to the manufacturer’s instructions.

### Statistical analysis

Two-sample *t* test was used to compare the percent of stem cells at 1 week of age to the percent of stem cells of the following mice ages: 3, 4, 8, 12, and 24 weeks. Two-sample *t* test was used again to compare the percent of stem cells of pre-sexual mice and sexually mature mice. *P* value of less than 0.05 was adopted for statistical significance.

## Results

### Identification and quantification of myometrial stem cells

**Fig. 1:**
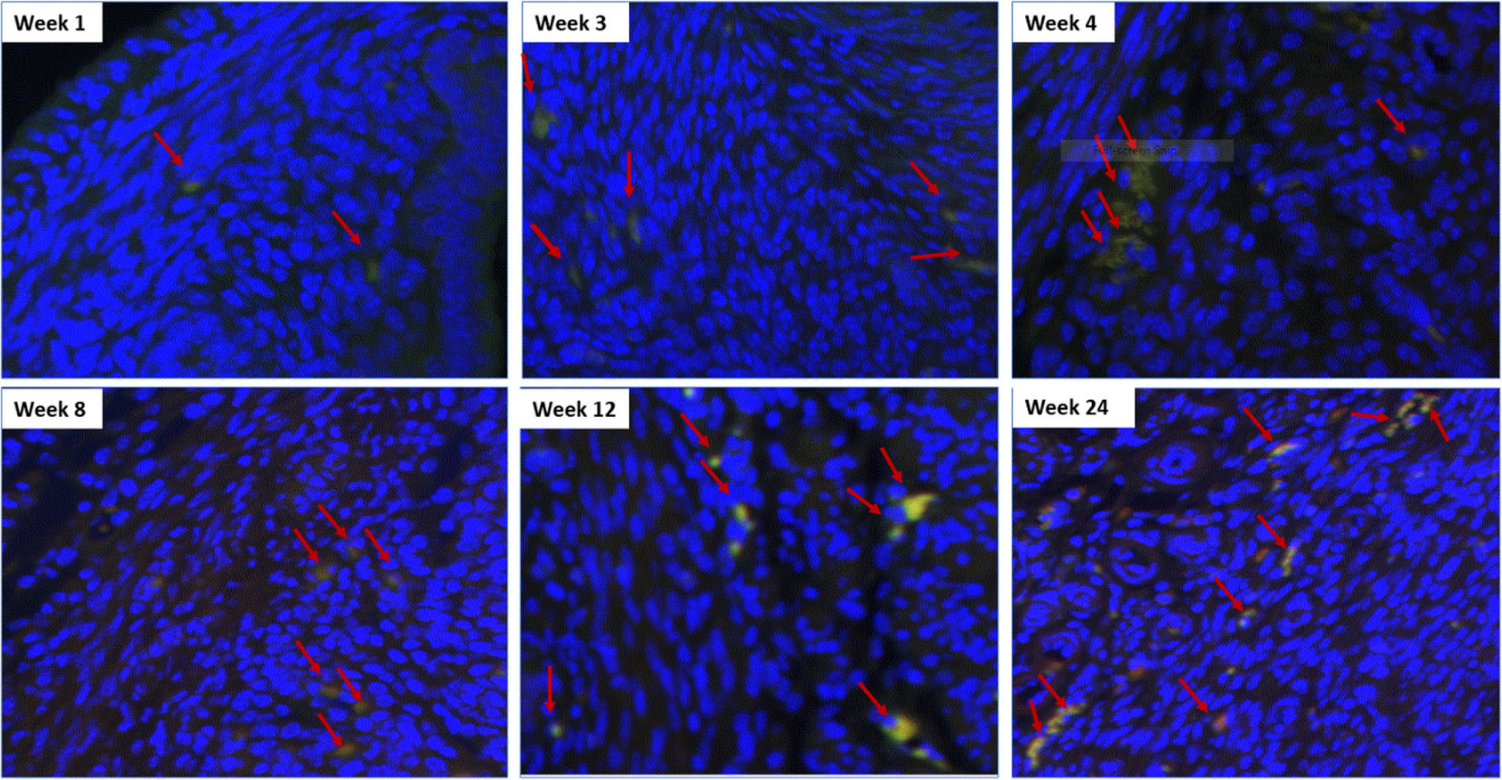
OCT4/GFP and CD44 co-staining of mice myometrium. Uterine ages 1, 3, 4, 8, 12, and 24 weeks (40×) are shown. Because Oct-4 was tagged with GFP, the cells expressing Oct-4 emitted green fluorescence. The conjugated CD44 antibody expressed Texas Red Fluorescence. The combination of both Oct-4 and CD44 staining (red and green) is yellow. Here, we show the yellow staining that indicates co-localization of Oct4/GFP and CD44

**Fig. 2:**
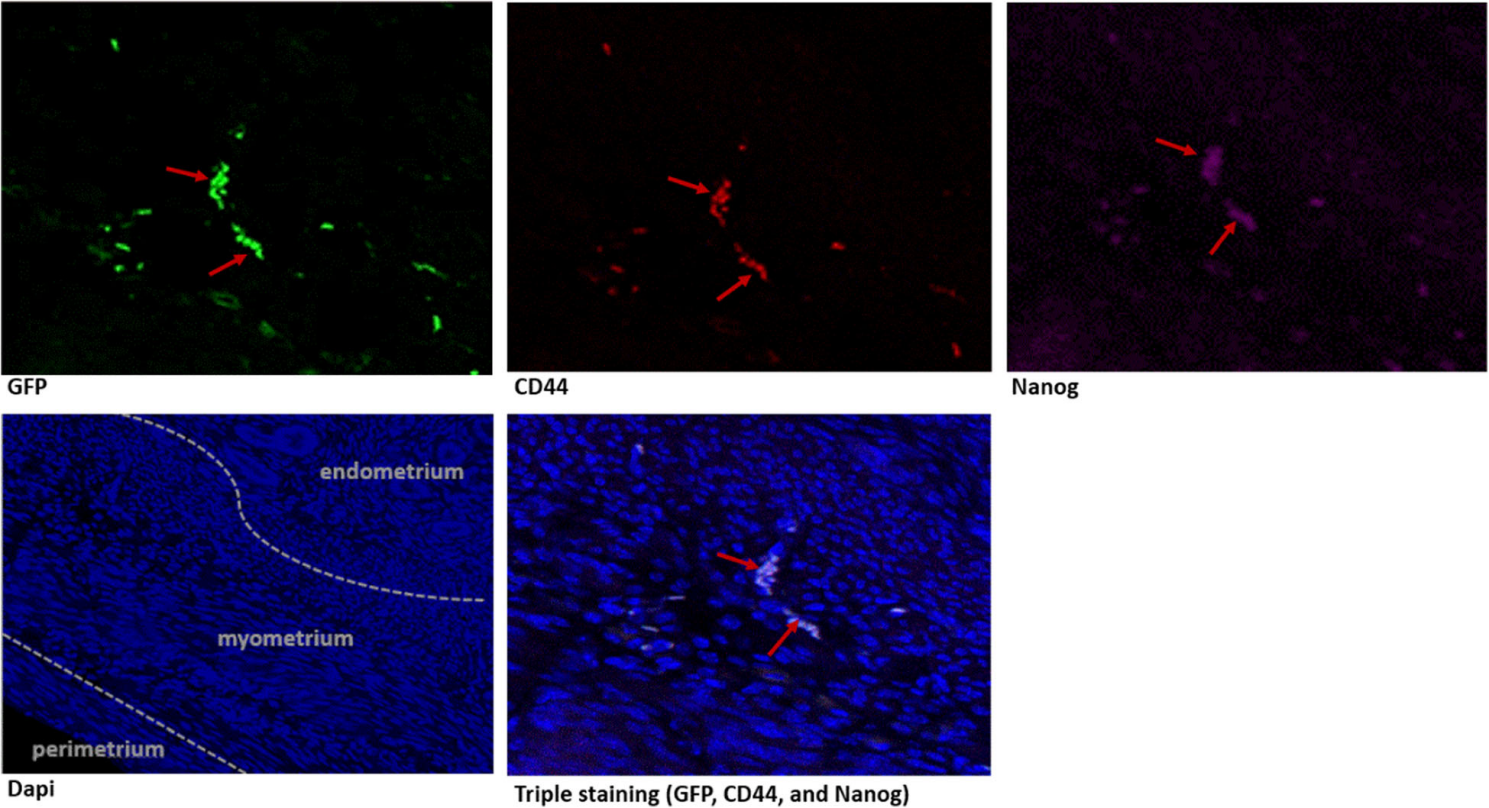
Myometrium triple staining with GFP, CD44, and Nanog of mice uterus at 24 weeks of age (20×). GFP: green fluorescence. CD44: red fluorescence. Nanog with 2nd antibody alexa fluor 647: purple fluorescence. DAPI: blue fluorescence

Because Oct-4 was tagged with GFP in this generalized transgenic mouse model, we could follow the expression of this primitive stem-cell marker with green fluorescence. Under low- and high-power magnification (20–40×), we were able to visualize Oct-4-expressing cells in the mouse myometrium. Then, to co-localize the Oct-4-positive cells with other well-known stem cells markers, immunofluorescence approaches were performed. The expression of the myometrial stem marker CD44 was evaluated using conjugated CD44 antibody. Because Oct-4 was tagged with GFP, the cells expressing Oct-4 emitted green fluorescence. The conjugated CD44 antibody expressed Texas Red Fluorescence. Thus, the combination of both Oct-4 and CD44 staining (red and green) is yellow, as demonstrated in Fig. 1. Figure 2 shows the added triple staining with Nanog at 24 weeks of age. The Nanog co-localizes with both Oct4 and CD44 confirming the stemness of the identified cells. We were unable to use Stro1 as an additional marker for mouse stem cells, as we previously described in human and rat myometrium [8], because Stro1 mouse Ab is not yet available. We then proceeded with evaluation of number of Oct-4+/Nanog+/CD44+ cells in uteri from mice 1, 3, 4, 8, 12, and 24 weeks of age. NIH ImageJ was used to count myometrium stem cells and to determine stem cell average for each uterine age as described in the method section.

**Fig. 3:**
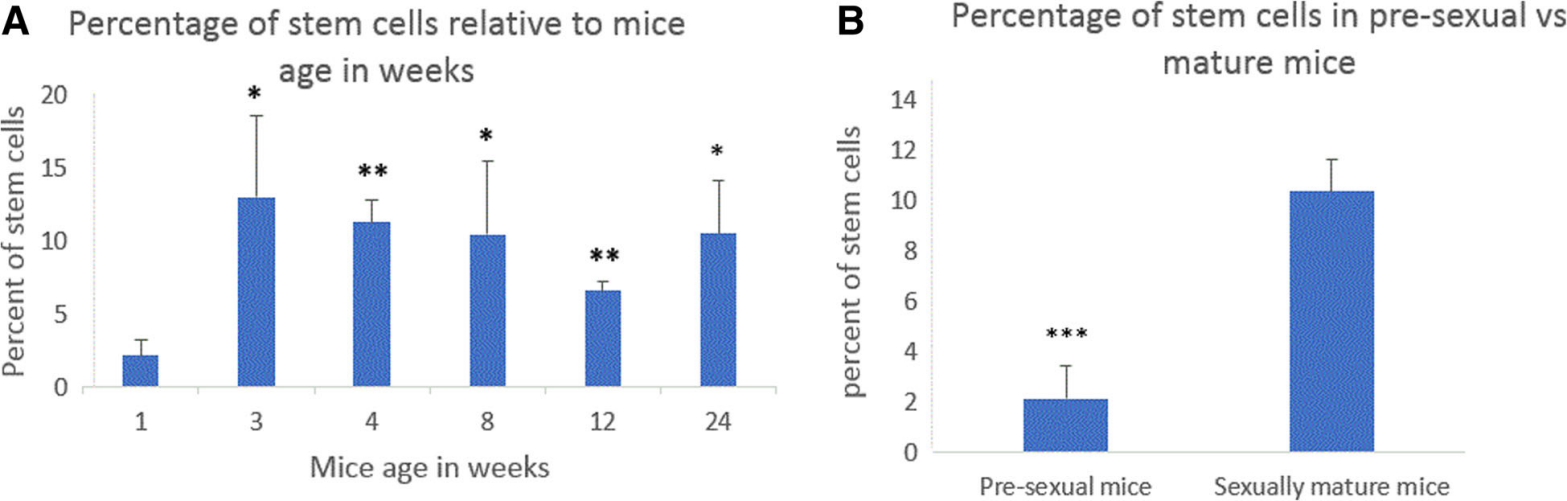
Percent of stem cells relative to uterine age. **a** *t* test statistical analysis, the percent of stem cells in 1 week mice was compared to 3, 4, 8, 12, and 24 week mice; *p* values were 0.01, 0.004, 0.02, 0.0028, and 0.0160, respectively. **b** Percent of stem cells in pre-sexual mice at 1 week of age was compared to percent of stem cells of sexually mature mice (average of stem cells in mice of age 3, 4, 8, 12, and 24). *P* value was 0.0003

The quantity of Oct-4+/CD44+/Nanog+ triple positive myometrial stem cells was significantly lower (2.14% ± 1.30) at 1 week of age as compared to all other tested older ages (*P* < 0.001). The quantity of myometrial stem cells at ages 3, 4, 8, 12, and 24 weeks was 13.01% ± 5.63, 11.30% ± 1.52, 10.43% ± 5.09, 6.60% ± 0.63, and 10.48% ± 3.65, respectively (Fig. 3). Our results clearly showed that at the pre-sexual age of 1 week, myometrial stem cells are significantly lower in frequency as compared to the sexually mature ages of 3 weeks and beyond. Our prior work [17] and others [24] demonstrated that human myometrial stem cells lack ER/PR expression and rely for their estrogen/progesterone responsiveness in adult myometrium on paracrine signaling from surrounding fully-differentiated ER/PR expressing myometrial cells. Consequently, we wanted to evaluate if the paucity of MSCs in young (1 week aged) myometrium is due to lack of ER/PR expression in surrounding differentiated myometrial cells or indeed due to lack of circulating estrogen and progesterone hormones at this early pre-sexual age, or both.

### Histological analysis of mouse myometrium for expression of estrogen and progesterone receptors

**Fig. 4:**
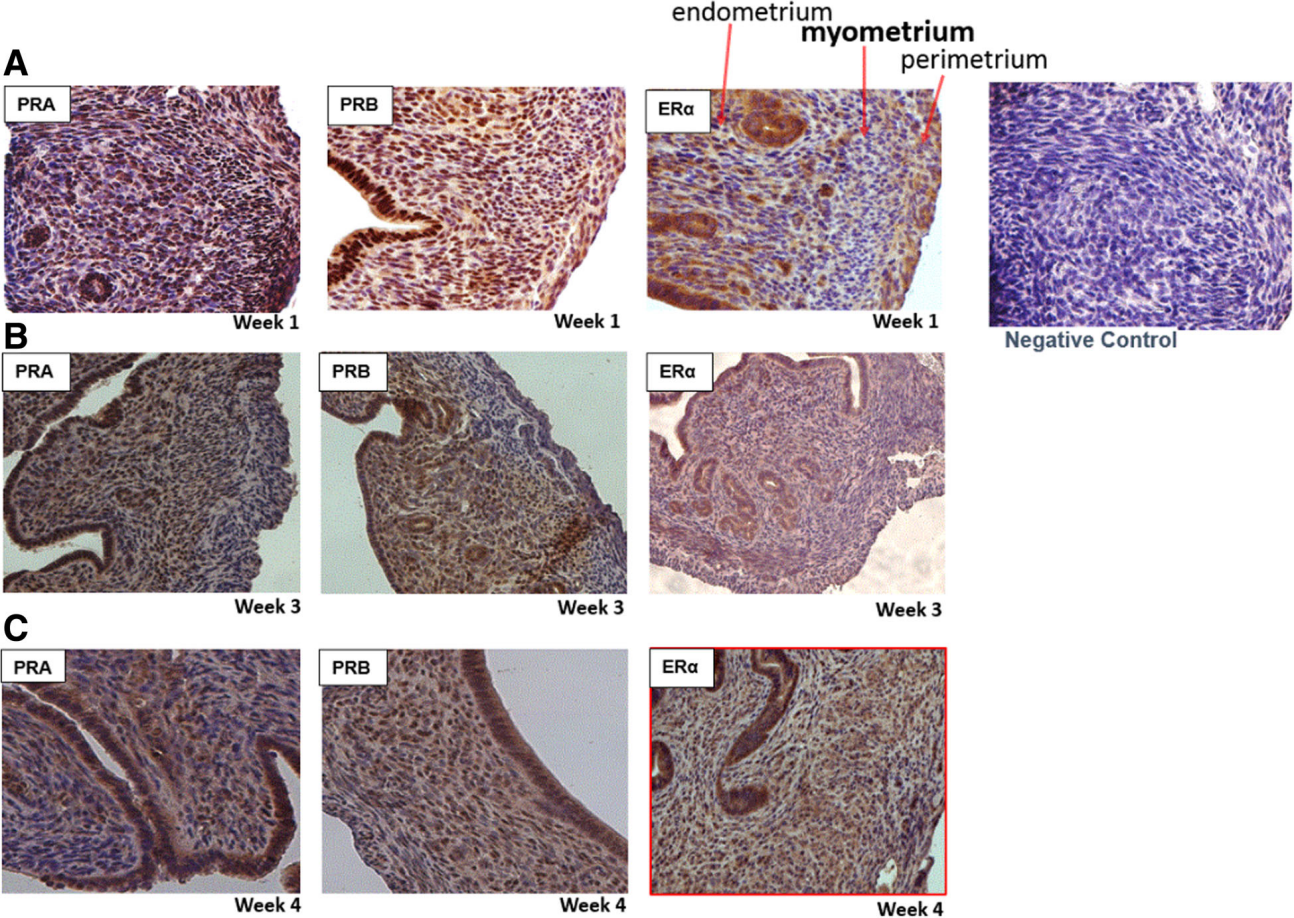
ERα and PR A&B staining in the myometrium of mice at 1, 3, and 4 weeks of age. **a** Week 1 myometrium staining of ER ERα and PR A&B (20×). **b** Week 3 staining of ER ERα and PR A&B (20×). **c** Week 4 staining of ER ERα and PR A&B (20×). Note negative control

To further investigate the low frequency of myometrial stem cell in pre-sexual mice, estrogen and progesterone receptor staining were carried out on samples at different age points. Interestingly, comparable and abundant positive staining for ERα and PR A&B receptors was detected in the myometrium of all tested ages: 1, 3, and 4 weeks (Fig. 4). As shown in Fig. 4, ERα and PR A&B staining in the myometrium of mice at ages 1, 3, and 4 weeks was evident in majority of myometrial smooth muscle cells and also in endometrium.

## Discussion

During pregnancy, the human uterus undergoes a 500- to 1000-fold increase in volume and a 24-fold increase in weight, which demonstrates the remarkable capacity of the myometrium to regenerate and remodel itself [3]. Although the regulation of myometrial functions during pregnancy and labor is mainly the result of the integration of endocrine and mechanical signals, it is reasonable to assume that the impressive myometrium regenerative ability must be tightly connected to a resident somatic stem cell population [3, 18]. There is increasing evidence that adult stem cells not only reside in highly regenerative tissue like bone marrow [25, 26], intestine, and epidermis [27, 28] but are also found in most tissue where they function to maintain hemostasis by replacing cells lost by apoptosis [2].

Nevertheless, stem cell research in uterine myometrium and leiomyomas is a relatively new area of inquiry, and few original articles have been published addressing this important cell population. In the last few years, several studies using the 5-bromo-2′-deoxyuridine and side population methods in murine and human myometrium have suggested the presence and functional relevance of somatic stem cells in this tissue [3, 23, 29]. These myometrial somatic stem cells lack smooth muscle cell markers and can be induced to differentiate into adipogenic and osteogenic lineages in addition to differentiating into smooth-muscle cells [12–14]. However, the uniqueness, scarcity, and lack of distinctive morphological characteristics, such as defining cell surface markers, make their identification and location a very complex task in most tissues including myometrium.

Although in our previous study we demonstrated the expression of Stro-1/CD44 in human and rat myometrium [17–19], here and for first time, we have identified the myometrial stem cell niche in the mouse myometrium by using the expression of CD44 and co-staining with other well-known stemness markers such as Oct-4 and Nanog. Moreover, we found that the percentage of Oct-4/CD44/Nanog myometrial stem cells was significantly lower at the pre-sexual age of 1 week than at the sexually mature ages of 3 weeks and older. Based on these results, we hypothesized that the very low frequency of Oct-4/CD44/Nanog myometrial stem cells, observed in the youngest mice, could be related to the lack of estrogen and progesterone sex hormones [30, 31]. Puberty onset is dependent on activation of the hypothalamic pituitary gonadal axis, which leads to gonadal steroid hormone production [32].

Myometrial smooth muscle cells have receptors for progesterone and estrogen, and they could play an important role in upregulating the proliferation of Oct-4/CD44/Nanog myometrial stem cells in murine myometrium, especially during pregnancy, via paracrine pathway [18]. Here, we demonstrated that both ER and PR are indeed abundantly expressed in the murine myometrium at ages 1, 3, and 4 weeks. This highly suggests that the low number of MSCs at 1 week of age is primarily due to lack of circulating estrogen and progesterone in these sexually immature mice [33, 34]. We have previously demonstrated that human and rat myometrial stem cells under-expressed ER and PR [19]; thus, it is likely that surrounding differentiated myometrial cells promote MSC proliferation in a paracrine manner, similar to human and rat myometrium [18]. This model has indeed been validated by recent work from Dr. Bulun’s group [24]. Consequently, the significantly lower frequency of stem cells in the pre-sexual age of 1 week is likely due to the lack of the estrogen and progesterone hormone ligand at that early age rather than the unavailability of the steroid receptors, which are similarly expressed, in neonatal and adult myometrium. Unfortunately, the serum levels of estrogen and progesterone were not available for the purchased transgenic mice; however, the chronological changes in mouse serum estrogen and progesterone are well established in the literature [33–37]. The role of estrogen in the onset of puberty and maintenance of reproduction is well established. The lowest serum levels of estradiol and progesterone were noted in prepubertal and ovariectomized mice in a hormonal analysis study [35]. Thus, our results suggest that stem cells are steroid dependent and increase in numbers with reproductive maturity at around 3 weeks of age in mice. Importantly, this data also emphasizes the vulnerability of neonatal myometrium to environmental xeno-estrogen and other chemical endocrine disruptor exposure. It is well established that exposure to xeno-estrogens during the sensitive developmental period increases risk of disease development later in life [38]. Our recent reports suggest that exposure to these environmental estrogens could lead to permanent reprogramming of myometrial stem cells and hence lead to adult onset of diseases such as uterine fibroids [19, 39]. However, further animal studies are needed to better understand the interplay between differentiated and myometrial stem cells as well as the various downstream pathways.

## Conclusion

In summary, our results suggest that the Oct-4/CD44/Nanog can be used as cell surface markers to identify a subpopulation of murine myometrial cells, exhibiting features of stem/progenitor cells. Furthermore, our results suggest that myometrial stem cells are sex steroid hormone dependent, likely via paracrine pathway, and increase in numbers with reproductive maturity and rise in serum estrogen and progesterone levels around 3 weeks of age in mice. The abundance and early-onset expression of ER/PR emphasize the vulnerability of neonatal myometrium to environmental endocrine disruptors that can potentially lead to permanent reprograming and adult onset of myometrial disorders such as uterine fibroids. These findings could offer a useful tool in better understanding the endocrinology of uterine function, providing novel insights into murine myometrial physiology as well as the origin of myometrial disorders such uterine fibroids.

## Additional file

Additional file 1: **Table S1.** List of antibodies used for immunohistochemistry staining. (PNG 36 kb)

## Abbreviations

ERα: Estrogen receptor α
MSCs: Mesenchymal stem cells
PR A&B: Progesterone receptors A and B
SSCs: Somatic stem cells
