# Supplementary figures and images for: The ontogeny of myometrial stem cells in OCT4-GFP transgenic mouse model
Source: Stem Cell Res Ther. 2018 Nov 29;9:333. doi: 10.1186/s13287-018-1079-7 (PMC6264618; doi:10.1186/s13287-018-1079-7)

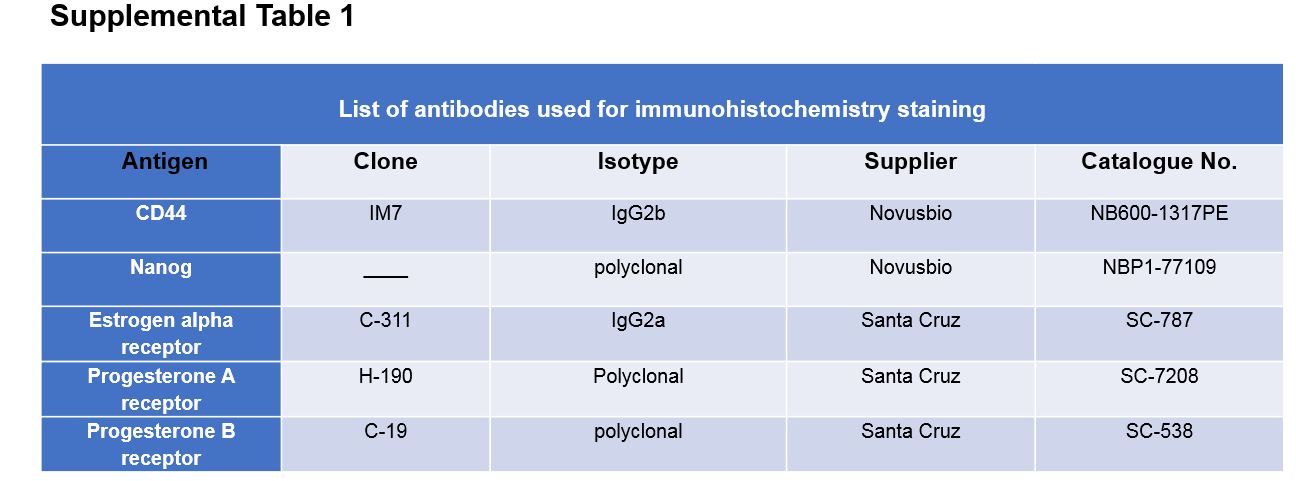

Supplement: Supplementary file 1 — Table S1. List of antibodies used for immunohistochemistry staining. (PNG 36 kb) [file 13287_2018_1079_MOESM1_ESM.png]
